# Effects of Tocotrienol-Rich Fraction Supplementation in Patients with Type 2 Diabetes: A Systematic Review and Meta-Analysis of Randomized Controlled Trials

**DOI:** 10.1016/j.advnut.2023.06.006

**Published:** 2023-06-14

**Authors:** Sonia C.W. Phang, Badariah Ahmad, Khalid Abdul Kadir, Uma Devi M Palanisamy

**Affiliations:** Jeffrey Cheah School of Medicine and Health Sciences, Monash University Malaysia, Bandar Sunway, Selangor, Malaysia

**Keywords:** tocotrienols, tocotrienol-rich fraction, diabetes mellitus, HbA1c, blood pressure, Hs-CRP

## Abstract

There are a large number of studies that have reported benefits of tocotrienol-rich fraction (TRF) in various populations with different health status. To date, no systematic reviews have examined randomized controlled trials (RCTs) on the effect of TRF supplementations specifically in patients with type 2 diabetes mellitus (T2DM). This systematic review and meta-analysis aim to examine the changes in HbA1c (glycated hemoglobin), blood pressure, and serum Hs-CRP (C-reactive protein high sensitivity) levels at post-TRF supplementation. Online databases including PubMed, Scopus, OVID Medline, and Cochrane Central Register of Controlled Trials were searched from inception until March 2023 for RCTs supplementing TRF in patients with T2DM. A total of 10 studies were included in the meta-analysis to estimate the pooled effect size. The Cochrane Risk-of-Bias (RoB) Assessment Tool was utilized to evaluate the RoB in individual studies. The meta-analysis revealed that TRF supplementation at a dosage of 250–400 mg significantly decreased HbA1c (−0.23, 95% CI: −0.44, −0.02, *P* < 0.05, *n* = 754), particularly where the intervention duration is less than 6 mo (−0.47%, 95% CI: −0.90, −0.05, *P* < 0.05, *n* = 126) and where duration of diabetes is less than 10 y (−0.37, 95% CI: −0.68, −0.07, *P* < 0.05, *n* = 83). There was no significant reduction in systolic and diastolic blood pressure and serum Hs-CRP (*P* > 0.05). The present meta-analysis demonstrated that supplementing with TRF in patients with T2DM decreased HbA1c but does not decrease systolic and diastolic blood pressure and serum Hs-CRP.

**Statement of Significance**

This is the first systematic review and meta-analysis that provides a comprehensive review on the effects of supplementation of TRF on HbA1c, blood pressure, and Hs-CRP in patients with T2DM.

## Introduction

International Diabetes Federation estimated that 537 million adults (20–79 y old) are living with diabetes mellitus in 2021 [1]. This estimate is projected to rise to a staggering 783 million by 2045 [1]. Type 2 diabetes mellitus (T2DM) and its host of complications confer a significant burden of mortality and morbidity worldwide as well as economic burden due to high costs in its management. These complications, nephropathy, retinopathy, and neuropathy, are often presented upon diagnosis or developed later during the course of the disease. In recent times, the COVID-19 pandemic has placed T2DM individuals at a higher risk of developing severe symptoms and morbidity. The current treatment approach for T2DM is primarily focused on blood-glucose control and cardio centric approaches as well as preventing and detecting diabetic complications at an early stage [2]. Conversely, once these complications have clinically manifested, there are no available treatments to alleviate them, and they can be fatal.

Vitamin E has been extensively studied for its preventive properties such as having antioxidant [[3], [4], [5]], anticholesterolemic [[6], [7], [8]], anti-inflammatory [9,10], anticancer [11,12], neuroprotective [[13], [14], [15], [16]], and cardioprotective properties [10,17,18]. Vitamin E is classified into tocotrienols and tocopherols whereby both class of compounds are subdivided into 4 isomers, namely, alpha, beta, gamma, and delta tocopherols, and tocotrienols (α, β, γ, δ) [19]. Tocopherols and tocotrienols are present in varying compositions in plant-based oils (palm oil, rice bran oil, and annatto) and grains (wheat, oat, barley, rice, and rye) [20]. One of the most abundant sources of tocotrienols is in the vegetable oil derived from palm fruit, also referred to as tocotrienol-rich fraction (TRF) [21]. TRF contains a ratio of 30% tocopherols and a 70% mixture of tocotrienol isomers [21].

Tocotrienols exhibit biological activities far superior or not found in tocopherols. Structurally, tocopherols and tocotrienols differ by their presence of saturated and unsaturated isoprenoid side chains, respectively [20]. Structural differences may contribute to the superiority of tocotrienols over tocopherol in biological activities. The unsaturated bonds and shorter side chains of tocotrienols allow greater fluidity and an even distribution in the phospholipid bilayer [20]. Furthermore, α-tocopherol is preferentially retained by body tissues through α-tocopherol transfer protein (α-TTP) but tocotrienols are rapidly degraded to short-chain carboxychromanols and conjugated counterparts, which have been shown to possess better biological effects [22]. This characteristic suggests that tocotrienols are more efficient than α-tocopherol at scavenging peroxyl radicals due to a more effective interaction in membrane environments [22].

Besides, the growing in vitro and in vivo evidence of the beneficial effects of the TRF treatment in T2DM, there has been a number of randomized clinical trials (RCTs) investigating the effect of TRF consumption in T2DM and its complications. However, there have not been any systematic reviews or meta-analyses that synthesized these RCTs to provide valuable information pertaining to the dosage, duration, and frequency of use of TRF for its potential application against T2DM and its complications. We have since systematically reviewed and appraised through meta-analysis, all available RCTs on the effect of TRF supplementation on the change in HbA1c, blood pressure, and biomarkers of the patients with T2DM.

## Methods

### Study protocol

This systematic review was conducted according to the Preferred Reporting Items for Systematic Reviews and Meta-Analyses (PRISMA), and the protocol for this review was registered on PROSPERO (CRD42021278476) on 21 November 2021.

### Search strategy

A comprehensive search was performed to identify relevant studies in the following databases: PubMed, OVID Medline, Scopus, and Cochrane Central Register of Controlled Trials from inception until 3 March 2023. The full detailed search strategy for each database is presented in Supplemental Table 1. All identified studies were pooled into a single database and duplicate articles were excluded.

### Eligibility criteria

The studies were selected for inclusion based on the following criteria: *1*) RCTs; *2*) compared supplementation of tocotrienols with placebo; *3*) patients with T2DM and/or with microvascular complications i.e., diabetic nephropathy, neuropathy, and retinopathy; and *4*) study time frame of within 10 y; 2012–2023.

Exclusion criteria were *1*) in vitro or animal studies or nonrandomized single-arm studies; *2*) α-tocopherol, nonspecified contents of vitamin E, tocotrienol supplementation that combined with other components; *3*) patients with chronic diseases i.e., cardiovascular diseases or cancer; *4*) abstracts, conference proceedings, and letters; and *5*) non-English studies. Duplicate publications were screened (articles based on the same dataset) through the trial registration number, list of authors, and patients’ baseline characteristics.

### Data extraction

One investigator SCWP performed the search in the databases. Citations were managed by EndNote X9.2 and relevant studies were imported into Covidence [23]. The title and abstract were screened and reviewed by SCWP and UDMP, a second investigator. Full texts of potential studies were screened based on the inclusion and exclusion criteria. Any conflicts between the investigators were resolved by BA, a third independent investigator. After identifying the relevant studies, the following data were extracted: study characteristics (country, sample size, and design), subject’s characteristics (age, sex, and duration of T2DM), intervention (type, contents, dosage, duration, frequency, and placebo), study outcomes, mean and SD of result measures at start, end of study, and/or changes in outcome measures from start to the end of the study.

### Quality assessment

Risk of bias (RoB) of all included studies was assessed independently by SCWP and UDMP using the Cochrane RoB Assessment Tool. The quality evaluation was performed on 6 domains: selection bias (random sequence generation and allocation concealment), performance bias (blinding of patients and personnel), detection bias (blinding of outcome data), attrition bias (incomplete outcome data), reporting bias (selective reporting), and other biases. Any conflicting opinions were settled by discussion.

### Statistical analyses

Meta-analyses were performed for HbA1c, blood pressure, and Hs-CRP, a biomarker of inflammation. The weighted mean differences with corresponding 95% CIs were determined by pooling eligible studies for meta-analyses. The heterogeneity among the studies was assessed using the chi-squared and *I*^*2*^ statistics, whereby a *P* value *<*0.1 and *I*^2^ above 50% indicates having significant heterogeneity, and a random effect model was used. Subgroup analyses were performed to explore the potential source of heterogeneity among studies, according to intervention duration (<6 mo and 6 mo), duration of diabetes (<10 y and >10 y), and baseline of HbA1c (<8.0% and >8.0%). A sensitivity analysis was performed. Funnel plots were used to visually inspect the presence of publication bias.

## Results

### Literature search

**FIGURE 1:**
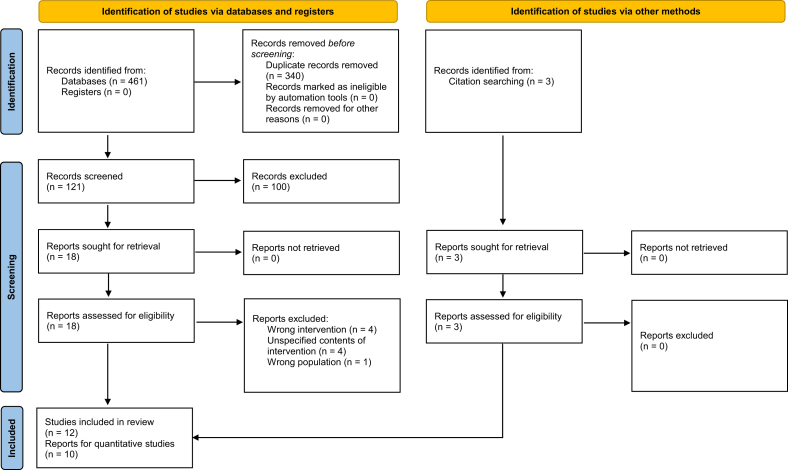
PRISMA statement.

The PRISMA chart presented in Figure 1 describes the selection process and the references retrieved from the database. The search identified 156 potentially relevant articles from PubMed, OVID Medline, Scopus, and Cochrane Central Register of Controlled Trials databases. Following the exclusion of duplicates and irrelevant articles, a total of 19 studies were selected for full text screening. Subsequently, 12 studies met the eligibility criteria for the current systematic review. Out of the 12 studies included, there were 2 groups (5 studies) involving patients from similar datasets, but these studies were included as each study reported unique parameters.

### Study characteristics

Majority of the 12 studies were carried out in Malaysia (*n* = 8) [[24], [25], [26], [27], [28], [29], [30], [31]] among other countries such as Australia (*n* =1) [32], Iran (*n* = 2) [33,34], and Pakistan (*n* = 1) [35]. The study sample size ranged from 43 to 300 patients. The mean age of the patients ranged from 50 to 70 y old. All of the studies recruited patients with T2DM either with or without microvascular complications such diabetic nephropathy [25,27], neuropathy [24,28] and retinopathy [26,29]. The duration of T2DM ranged from 3 to 18 y. The intervention duration ranged from 8 wk to 18 mo. Generally, the studies used palm oil-derived TRF as the intervention, with only one study that used annatto-derived tocotrienols [35]. Majority of the study interventions are tocotrienols supplemented in the form of a capsule whereas 2 studies used canola oil supplemented with tocotrienols, as an addition to cooked foods or salad [33,34]. Most studies used a placebo that is essentially devoid of the tocotrienols or low in tocotrienols. If the formulation is in a capsule, the placebo’s appearance is of the same size, shape, and color. Seven studies used sunflower oil in the capsule form, 2 studies used pure canola oil as an addition to salad, one study used a cellulose capsule, one study used a tocotrienol-free palm oil capsule whereas one study used capsules of palm olein devoid of carotenes and low in vitamin E. The dosage of TRFs ranged from 200 to 420 mg total tocotrienols. A total of 10 studies were selected for meta-analysis, studies having the same cohort but different study durations were excluded. The characteristics of the studies and patients included in the systematic review are presented in Table 1.

**TABLE 1:** Characteristics of studies included in the systematic review

Author and y	Country	Population health status	Sample size		Sex (M/F)		Mean age (y)		Duration of diabetes (y)		BMI (mg/kg)		Duration of study (wk)	T3 dosage/composition/source	Placebo
			T	P	T	P	T	P	T	P	T	P			
Haghighat et al., 2014 [33]; Vafa et al., 2015 [34]	Iran	T2DM	23	22	5 M, 18 F	7 M, 15 F	55.9 ± 5.9	55.2 ± 5.6	4.8 ± 4.1	4.7 ± 2.9	25.1 ± 6.9	26.1 ± 3.3	8	200 mg T3-enriched canola oil/69.3 mg α-T3, 87.15 mg γ-T3/palm-based	Pure canola oil
Stonehouse et al., 2016 [32]	Australia	T2DM	28	29	18 M, 10 F	18 M, 11 F	60.5 ± 12.0	61.0 ± 10.5	5.5 ± 11.6	2.5 ± 5.9	33.3 ± 4.8	32.4 ± 4.3	8	420 mg T3-rich vitamin E capsule/135.2 mg α-T3, 19.4 mg β-T3, 195.4 mg γ-T3, 70 mg δ-T3/palm oil	Palm olein devoid of carotenes and low in vitamin E capsule
Hor et al., 2018 [24] 1	Malaysia	T2DM with diabetic neuropathy	150	150	67 M, 83 F	63 M, 87 F	58.0 (8.9)	57.2 (8.9)	11.8 (7.6)	11.1 (8.0)	27.6 ± 5.4	28.2 ± 5.1	52	400 mg T3-rich vitamin E capsule/123.04 mg α-T3, 225.6 mg γ-T3, 51.36 mg δ-T3/palm oil	T3-free palm oil capsule
Tan et al., 2018 [25] 1^,^^2^	Malaysia	T2DM with diabetic nephropathy	22	23	16 M, 6F	15 M, 8F	59.9 ± 10.2	63.3 ± 10.4	18.2 ± 10	17.9 ± 7.7	29.4 ± 5.4	29.3 ± 4.7	8	400 mg T3-rich vitamin E capsule/123.04 mg α-T3, 225.6 mg γ-T3, 51.36 mg δ-T3/palm oil	Sunflower oil capsule^3^
Tan et al., 2019 [30] 1^,^^2^	Malaysia	T2DM with diabetic nephropathy	27	27	18 M, 9F	17 M, 10 F	59.0 ± 10.0	62.8 ± 11.6	20.7 ± 9.9	16.2 ± 8.1	29.4 ± 5.4	29.3 ± 4.7	12	400 mg T3-rich vitamin E capsule/123.04 mg α-T3, 225.6 mg γ-T3, 51.36 mg δ-T3/palm oil	Sunflower oil capsule^3^
Ng et al., 2020 [31] 1^,^^2^	Malaysia	T2DM with diabetic neuropathy	39	41	25 M, 14 F	27 M, 14 F	63.0 ± 12.0	64.0 + 15.0	14.0+ 10.0	13.0 + 11.0	28.0 ± 4.2	28.4 ± 5.1	8	400 mg T3-rich vitamin E capsule/123.04 mg α-T3, 225.6 mg γ-T3, 51.36 mg δ-T3/palm oil	Sunflower oil capsule^3^
Mahjabeen et al., 2020 [35]	Pakistan	T2DM	55	55	31 M, 24 F	36 M, 19 F	52.5 ± 14.0	50.0 ± 12.6	9.0 ± 3.8	8.5 ± 4.6	29.2 ± 3.9	28.5 ± 2.7	24	250 mg δ-tocotrienol capsule/90% δ-T3, 10% γ-T3/annatto	Cellulose capsule
Koay et al., 2021 [27] 1^,^^2^	Malaysia	T2DM with diabetic nephropathy	31	28	20 M, 11 F	18 M, 10 F	66 (13)	70 (13)	15.3 ± 7.6	17.9 ± 8.9	28.1 ± 4.4	29.1 ± 5.0	52	400 mg T3-rich vitamin E capsule/123.04 mg α-T3, 225.6 mg γ-T3, 51.36 mg δ-T3/palm oil	Sunflower oil capsule^3^
Chiew et al., 2021[26]^1^^,^^2^	Malaysia	T2DM with diabetic retinopathy	21	22	17 M, 4 F	15 M, 7 F	59.8 ± 7.6	63.1 ± 8.6	17.8 ± 9.4	18.8 ± 7.2	28.1 ± 4.2	27.6 ± 4.2	8	400 mg T3-rich vitamin E capsule/123.04 mg α-T3, 225.6 mg γ-T3, 51.36 mg δ-T3/palm oil	Sunflower oil capsule^3^
Chuar et al., 2021 [28] 1^,^^2^	Malaysia	T2DM with diabetic neuropathy	43	45	29 M, 14 F	29 M, 16 F	63 (11.5)	64 (13)	15.5 ± 8.6	16.2 ± 9.1	28.0 ± 4.1	28.3 ± 5.1	52	400 mg T3-rich vitamin E capsule/123.04 mg α-T3, 225.6 mg γ-T3, 51.36 mg δ-T3/palm oil	Sunflower oil capsule^3^
Ho et al., 2022 [29] 1^,^^2^	Malaysia	T2DM with diabetic retinopathy	27	28	16 M, 11 F	16 M, 12 F	62.9 ± 9.6	62.0 ± 7.7	18.3 ± 8.7	16.6 ± 7.7	27.6 ± 5.4	28.2 ± 5.1	52	400 mg T3-rich vitamin E capsule/123.04 mg α-T3, 225.6 mg γ-T3, 51.36 mg δ-T3/palm oil	Sunflower oil capsule^3^

Abbreviations: T2DM, type 2 diabetes mellitus; M, male; F, female; T, tocotrienol-rich fraction; P, placebo; T3, tocotrienols.

1 Multicenter, study was conducted in multiple locations within the same country.

2 The studies are of the same population investigating different parameters.

3 The authors have been contacted on the source of the placebo.

**FIGURE 2:**
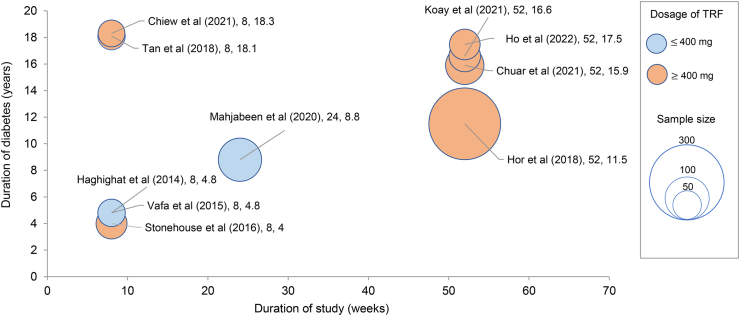
Bubble chart showing duration of diabetes plotted against duration of study, dosage of tocotrienols, and sample size.

The bubble chart describes the studies based on its duration of study, dosage of tocotrienols, and sample size against duration of diabetes (Figure 2). The size of the bubbles denotes the sample population whereas the color of the bubbles corresponds to the dosage of the tocotrienols. Majority of the studies included patients with duration of T2DM over 10 y with a population size of 80 patients and below which had a study duration of 52 wk and an intervention dosage of more than 400 mg total tocotrienols [[24], [25], [26], [27], [28], [29]]. Four studies had a study duration of 8 wk with both a low and high (< 10 y and >10 y) duration of diabetes and mixed tocotrienol dosage.

### Adverse events

Five RCTs did not report any significant adverse events (AE) in both intervention and control groups. In Koay et al. [27] and Chuar et al. [28], a total of 19 patients reported at least one AE throughout the study period citing gastrointestinal disorders, whereas 5 of them reported an SAE, seizure, viral fever, and/or renal stone removal, tumor removal, and vaginal hysterectomy. Three patients withdrew due to AEs. Hor et al. [24] reported 7 AEs in the control group and 12 AEs in the tocotrienols group. Stonehouse et al. [32] reported one AE in both the control and tocotrienol groups.

### Qualitative data assessment

**FIGURE 3:**
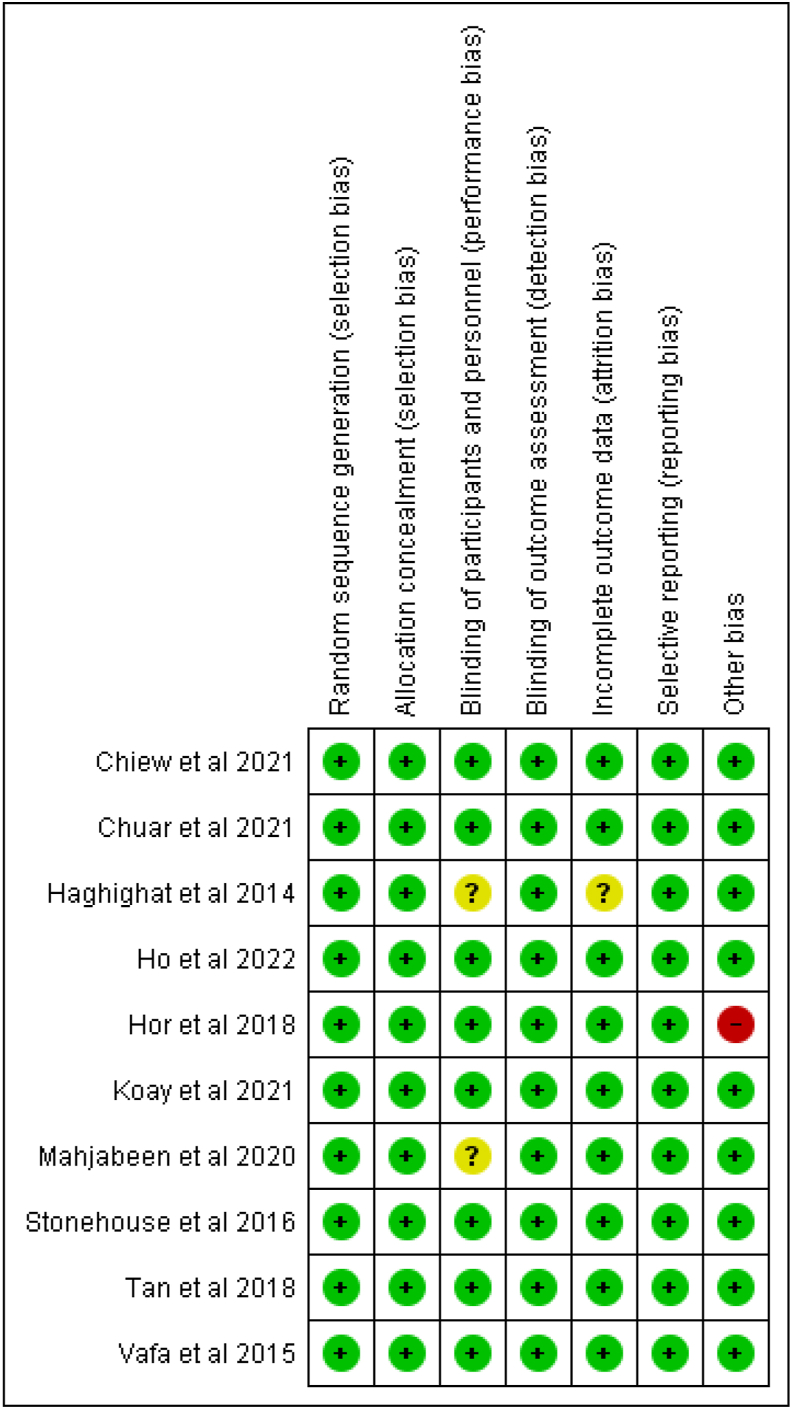
Risk of bias summary.

**FIGURE 4:**
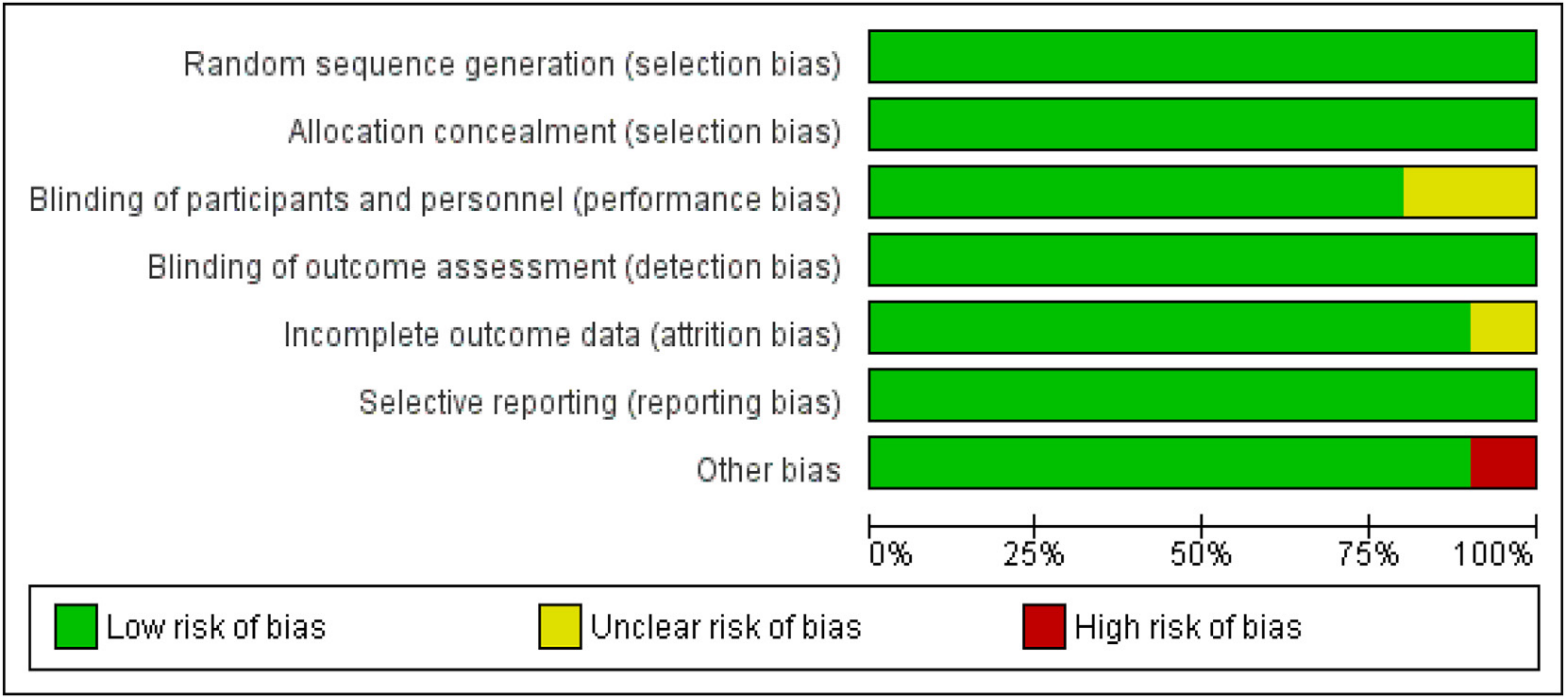
Risk of bias graph.

Cochrane RoB Assessment tool indicated that 7 studies were of high quality (Good) with a total low RoB for all domains of this tool (FIGURE 3, FIGURE 4) [[25], [26], [27], [28], [29],^32^,^34^]. Two studies had a moderate quality (Fair) in which one or more domains have an unclear RoB [33,35]. Only one of the studies had a high RoB for one domain [24].

### Publication bias

Visual inspection of the funnel plots investigating the effect of TRF supplementation on HbA1c demonstrated asymmetry (Supplemental Figure 1). This may not be due to the publication bias but could also be attributed to the statistical heterogeneity between the larger and smaller studies. For instance, Hor et al.’s [24] study had the largest number of participants (*n* = 300) as compared with the other smaller populations (*n* = 40–100). However, no publication bias was indicated for the effects of TRF on systolic blood pressure (Supplemental Figure 2) and diastolic blood pressure (Supplemental Figure 3). Further statistical analysis for the funnel plot asymmetry could not be performed when there are fewer than 10 studies as the power would be too low to distinguish true bias from chance [36].

### The effect of TRF supplementation on HbA1c

**FIGURE 5:**
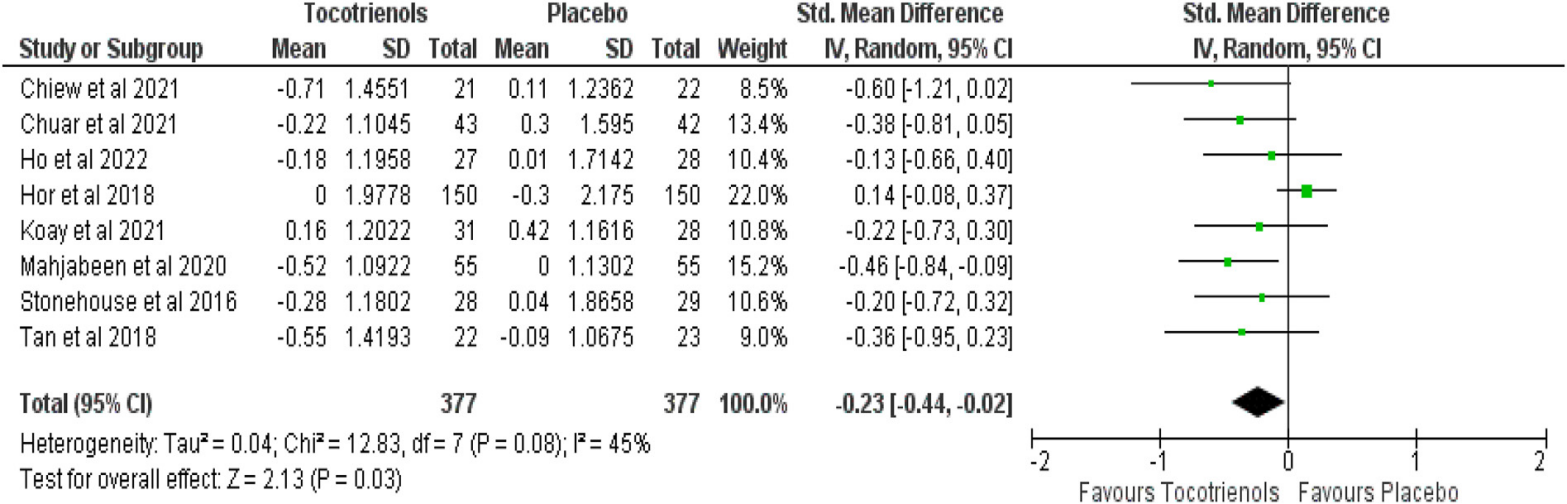
Effect of tocotrienol-rich fraction supplementation on HbA1c.

Eight of 10 studies comprising 754 patients (intervention group = 377, control group = 377) reported the effect of tocotrienols on HbA1c. Pooled results showed that tocotrienol supplementation caused a significant reduction in HbA1c (SMD: −0.23, 95% CI: −0.44, −0.02) *P* = 0.03) with moderate heterogeneity (*I*^*2*^ = 45%, *P* = 0.08) (Figure 5).

### The effect of TRF supplementation on blood pressure

**FIGURE 6:**
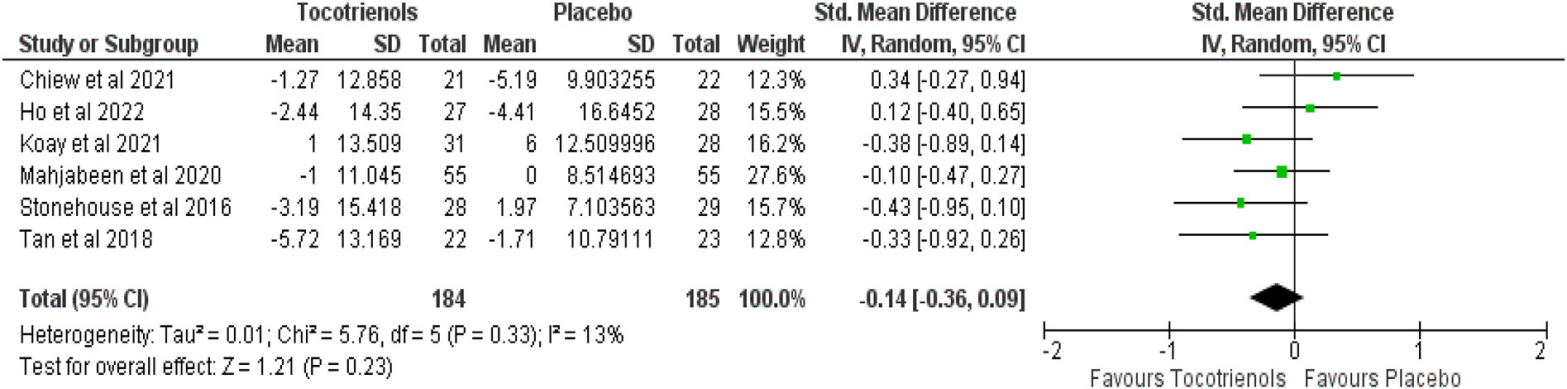
Effect of tocotrienol-rich fraction supplementation on systolic blood pressure.

**FIGURE 7:**
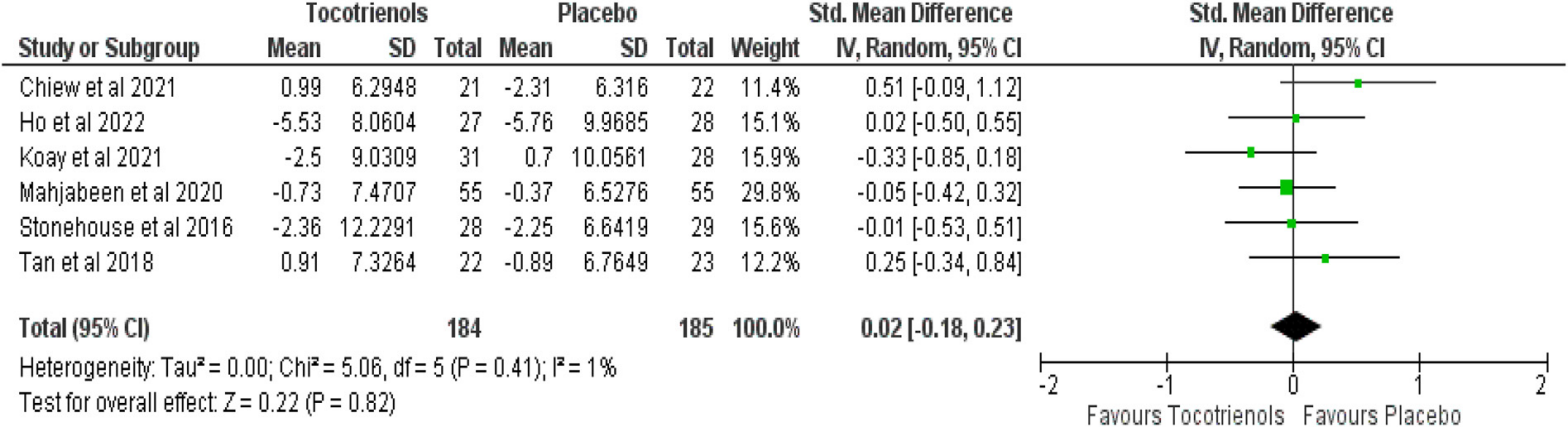
Effect of tocotrienol-rich fraction supplementation on diastolic blood pressure.

Six studies comprising 369 patients (intervention group = 184, control group = 185) presented the effect of TRF on systolic and diastolic blood pressure. Pooled results showed that tocotrienol supplementation caused a nonsignificant reduction in systolic blood pressure (SMD: −0.14, 95% CI: −0.36, 0.09, *P* = 0.23) with a small heterogeneity (*I*^*2*^ = 13%, *P* = 0.33) (Figure 6), and a nonsignificant difference in diastolic blood pressure (SMD: 0.02, 95% CI: −0.18, 0.23, *P* = 0.82) with a small heterogeneity (*I*^*2*^ = 1%, *P* = 0.41) (Figure 7).

### The effect of TRF supplementation on serum Hs-CRP levels

**FIGURE 8:**
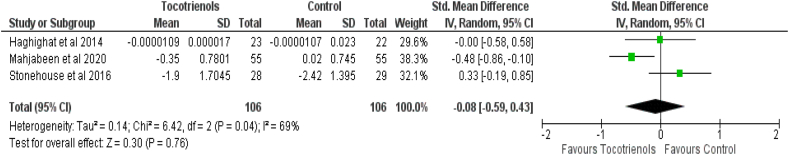
Effect of tocotrienol-rich fraction supplementation on serum Hs-CRP levels.

Three studies comprising 213 patients (intervention group = 106, control group = 107) presented the effect of tocotrienols on serum Hs-CRP levels. Pooled results showed that tocotrienol supplementation caused a nonsignificant reduction in serum Hs-CRP levels (SMD: −0.08, 95% CI: −0.59, 0.43, *P* = 0.76) with substantial heterogeneity (*I*^*2*^ = 69%, *P* = 0.04) (Figure 8).

#### Subgroup analyses

Subgroup analyses were performed to explore the potential source of heterogeneity among studies; according to intervention duration (<6 mo and 6 mo), duration of diabetes (<10 y and >10 y), and baseline of HbA1c (<8.0% and >8.0%). Findings from the subgroup analyses are outlined in Table 2. Subgroup analyses indicated significant reductions in HbA1c when the intervention duration was <6 mo, duration of diabetes <10 y, and a baseline HbA1c <8.0% with low heterogeneity. The studies reporting systolic blood pressure were further stratified on the basis of duration of diabetes. A similar nonsignificant reduction of systolic blood pressure was observed but with a low heterogeneity.

**TABLE 2:** Subgroup analyses of tocotrienols on HbA1c

	Number of studies	n	Mean difference (95% CI)	*P* within group	*P* heterogeneity	*I*^*2*^ (%)
HbA1c						
Pooled analysis	8	754	−0.23 (−0.44, −0.02)	0.03	0.08	45
Intervention duration						
<6 mo	4	126	−0.47 (−0.90, −0.05)	0.03	0.59	0
>6 mo	4	251	−0.09 (−0.36, 0.18)	0.50	0.15	44
Duration of diabetes						
<10 y	2	83	−0.37 (−0.68, −0.07)	0.02	0.42	0
v>10 y	6	294	−0.19 (−0.45, 0.06)	0.14	0.09	47
Baseline HbA1c						
<8.0%	4	129	−0.25 (−0.49, −0.00)	0.05	0.90	0
>8.0%	4	248	−0.27 (−0.68, 0.13)	0.19	0.01	73
	3^1^	98	−0.47 (−0.75, −0.19)	0.001	0.86	0
Systolic blood pressure						
Pooled analysis	6	369	−0.14 (−0.36, 0.09)	0.23	0.33	13
Duration of diabetes						
<10 y	2	83	−0.21 (−0.51, 0.09)	0.18	0.32	0
>10 y	4	101	−0.07 (−0.41, 0.26)	0.67	0.22	31

1 Sensitivity analysis was performed by removing this study by Hor et al., 2018 [24].

### Sensitivity analysis

A sensitivity analysis was performed by sequentially removing individual studies at a time. We found that when the study by Hor et al. [24], which had a high RoB and the largest sample size (*n* = 300), was excluded, it resulted in a higher reduction of HbA1c; (SMD: −0.34, 95% CI: −0.53, −0.16, *P* = 0.0003). No heterogeneity was detected (*I*^*2*^ = 0%, *P* = 0.90). For subgroup analysis, the exclusion of the trial demonstrated that patients on TRF supplementation with a higher baseline of HbA1c >8.0% had greater reductions of HbA1c when compared with those with a baseline HbA1c of <8.0%.

## Discussion

The management of T2DM encompasses the use of oral hypoglycemic drugs and insulin, which can effectively reduce blood-glucose levels [37]. However, these treatments have limitations as they may lose its effectiveness in glycaemic control over time as demonstrated by longitudinal studies; leading to hypoglycemia and weight gain [38]. These limitations have driven investigations into nonpharmacological treatments such as vitamin supplementation as potential adjuncts to existing treatments. A growing number of clinical trials have explored the affiliation of vitamin status in patients with T2DM, whereby antioxidant vitamins A, C, and E have been found to be reduced in patients with T2DM [39]. A reduction in plasma tocopherol has been reported in patients with T2DM with a longer disease duration, and in general populations, higher levels of α-tocopherol have been correlated with the decreased risk of diabetes [39]. The increasing number of RCTs investigating the effect of TRF supplementation in the T2DM population underscores the importance of this systematic review. To the best of our knowledge, this is the first systematic review and meta-analysis of RCTs to study the effects of supplementation of TRF on HbA1c, blood pressure, and Hs-CRP in patients with T2DM.

The meta-analysis from this study provides evidence in support of the association of supplementation of TRF with the significant reduction of HbA1c. The subgroup analyses revealed that TRF supplementation significantly decreased HbA1c in studies where the intervention duration is less than 6 mo (*n* = 126) and where duration of diabetes of the patients is less than 10 y (*n* = 83). A meta-analysis by Xu et al.[40] reported a more significant effect on HbA1c (−0.58%, 95% CI:−0.83 to −0.34) in studies with lower baseline serum vitamin E, which is also consistent with the findings of an earlier meta-analysis by Suksomboon et al. [41]. However, both meta-analyses mentioned above included study interventions of both tocopherols and tocotrienols, with only one study with tocotrienols alone.

Having performed the sensitivity analysis, we found that patients with a higher HbA1c baseline demonstrated a greater reduction of HbA1c post-intervention in comparison to patients with a low HbA1c baseline. Similar studies have been reported whereby in one study by Diamant et al. [37] reduction in HbA1c is generally highest when baseline HbA1c was high, regardless of the type of blood-glucose lowering treatment. Chen et al. [38] as well reported a near 1% reduction in HbA1c in the post-treatment group where the baseline HbA1c concentration was 8%. The benefit of treatment on glycaemic control was more significant in studies with a higher baseline HbA1c concentration than in studies with a lower baseline HbA1c concentration [38].

The milestone UKPDS study established the importance of glycaemic control in the prevention and minimization of diabetes complications in patients with diabetes [36,42]. The reduction of 1.0% HbA1C is associated with a significant reduction in cardiac mortality (24%), myocardial infarction (14%), microvascular complications (37%), and amputation and peripheral vascular disease (43%) [36,42]. The significant reduction of HbA1c is clinically important as it can minimize the micro- and macrovascular complications and cardiac deaths in patients with diabetes [36].

This review showed that TRF caused a reduction in systolic blood pressure; however, it is not significant. There was no effect on diastolic blood pressure. This may be attributed to heterogeneity in the duration of diabetes as the majority of the studies had patients with a longer diabetes duration; 16–18 y [[25], [26], [27],^29^] versus 3–9 y [32,35] in 2 studies. The subgroup analysis demonstrated that studies with populations with shorter duration of T2DM <10 y have a higher nonsignificant reduction in systolic blood pressure as compared with a longer duration of diabetes >10 y. There is one meta-analysis of 17 studies by Li et al. [43] that investigated the association of tocotrienols consumption with blood pressure. The review concluded that consumption of tocotrienols in patients with T2DM was affiliated with a nonsignificant reduction in diastolic blood pressure (SMD: 0.20mmHg, 95% CI: –0.09, 0.50, *P* = 0.034), and a nonsignificant reduction in systolic blood pressure (SMD: 0.19 mmHg, 95% CI: −0.53, 0.56, *P* = 0.947). The average duration of diabetes of the patients is <10 y. However, a study on tocotrienol supplementation, using spontaneously hypertensive rats (SHRs), showed favorable downregulation of lipid peroxidation, contributing to blood-pressure reduction effects in SHRs [44].

Many animal and human studies have discussed the potential roles of tocotrienols in the pathology of T2DM. Studies have shown that the biological activities of tocotrienols can be attributed to its potency in anti-inflammation, antioxidation, and cholesterol lowering activities. The association between T2DM and oxidative stress is well established; this is due to the fact that hyperinsulinaemia and hyperglycaemia enhances the generation of reactive oxygen species hence contributing to the rise in oxidative stress [45]. The effects of raising oxidative stress in T2DM includes impaired insulin signaling, β-cell function, and promote hemoglobin glycation. There are several meta-analyses that investigated the effects of vitamin E supplementation on inflammation and oxidative stress; however, these studies were primarily based on tocopherols and not tocotrienols. At present, there is one meta-analysis of 19 studies by Khor et al. [46] that reported that the tocotrienols supplementation at 400 mg/d may reduce MDA levels. The same study reported a significant reduction in C-reactive protein levels (WMD: −0.52 mg/L, 95% CI: −0.73, −0.32, *P* < 0.001) after tocotrienols supplementation but this finding was attributed to a single study using δ-tocotrienols, not mixed tocotrienols [46]. This systematic review could not provide clinical evidence on the anti-inflammatory and anti-oxidative nature of tocotrienols. There were only 3 studies that reported levels of MDA [[33], [34], [35]], CRP [[32], [33], [34]], and cystatin C [[25], [26], [27]], and only one study measured serum nitric oxide levels [33,34]. Pooled results showed that tocotrienol supplementation caused a nonsignificant reduction in serum Hs-CRP levels (SMD: −0.08, 95% CI: −0.59, 0.43, *P* = 0.76) with substantial heterogeneity (*I*^*2*^ = 69%, *P* = 0.04).

Despite the overwhelming evidence of in vivo and clinical studies suggestive of the effects of TRF in the reduction of HbA1c and systolic blood pressure in patients with T2DM, this systematic review could not provide sufficient evidence to support its use as an adjunct in the management of T2DM.

This systematic review has several limitations. There is moderate heterogeneity in our analysis on the effects of TRF supplementation on HbA1c and substantial heterogeneity for serum Hs-CRP levels. This may be due to variation in sample size, study design, formulation and dosage, duration of diabetes as well as the intervention duration. A random-effects model was used to take into account interstudy heterogeneity. Subgroup analyses were also conducted for intervention duration, duration of diabetes, and baseline HbA1c to explore whether the treatment effect varies across different levels of these factors. Another limitation of this review is that the number of studies investigating effects of TRF supplementation in specific diabetic complications (diabetic retinopathy, neuropathy, and nephropathy) were small. As such, meta-analysis on each diabetic complication could not be performed. Six of the 10 studies were conducted in Malaysia whereby majority consumes palm oil which is highly rich in tocotrienols on a regular basis. Hence, the results may not be generalizable to other populations due to different dietary intakes and different baseline levels of TRF. In addition, many studies did not report serum or plasma levels of tocotrienols; hence, it is not clear whether the effect is reliant on the status of serum tocotrienols. Thus, the baseline levels of tocotrienols and dietary habits should be taken into consideration in reporting. Majority of the included studies had a short-term follow-up period. Consequently, the ability to derive conclusions of the long-term implications of TRF supplementation is limited. Future studies with extended follow-up periods are necessary to gain a comprehensive understanding of long-term benefits and drawbacks of TRF.

This meta-analysis showed that supplementation of high dose TRF with a dosage between 250 and 400 mg significantly decreased HbA1c in patients with T2DM. The reduction is particularly significant at an intervention duration of <6 mo and the efficacy is enhanced if the duration of diabetes of the patients is <10 y. The significant reduction of HbA1c is clinically important as it can minimize the micro- and macrovascular complications and cardiac deaths in patients with T2DM. While the meta-analysis did not show a significant reduction in systolic and diastolic blood pressure, there is still potential for TRF to offer benefits in these areas. Future long-term randomized trials should be conducted to assess the effects of TRF in T2DM alongside the patients’ dietary habits and baseline levels of TRF. In addition, further research should investigate the effects of different isoforms of TRF and dosages in patients with T2DM. Overall, this meta-analysis offers valuable insights into the potential benefits of TRF supplementation in T2DM, highlighting the need for continued research in this area to optimize its clinical application and improve outcomes for patients.

## Author contributions

The authors’ responsibilities were as follows—SCWP, UDMP, KAK: designed the study; SCWP: developed the search strategy and extracted the data; SCWP, UDMP, BA: conducted full text screening; SCWP: conducted the analyses and drafted the manuscript; SCWP, UDMP: assessed the RoB of the meta-analyses and interpreted the results; SCWP wrote the manuscript; SCWP, UDMP, BA: revised the manuscript; and all authors: read and approved the final manuscript.

## Conflict of Interest

The authors report no conflicts of interest.

## Funding

The authors reported no funding received for this study.

## Data availability

The data used in this review are available from the corresponding author upon request.

## Appendix A Supplementary data

Multimedia component1 Multimedia component1

Multimedia component2 Multimedia component2
