# Supplementary material for: Effects of Tocotrienol-Rich Fraction Supplementation in Patients with Type 2 Diabetes: A Systematic Review and Meta-Analysis of Randomized Controlled Trials
Source: Adv Nutr. 2023 Jun 14;14(5):1159–69. doi: 10.1016/j.advnut.2023.06.006 (PMC10509396; doi:10.1016/j.advnut.2023.06.006)
Supplement: Multimedia component1 [file mmc1.pdf]

Title: Effects of tocotrienol-rich fraction supplementation in patients with type 2 diabetes: A systematic review and meta-analysis of randomised controlled trials

First author: Sonia CW Phang

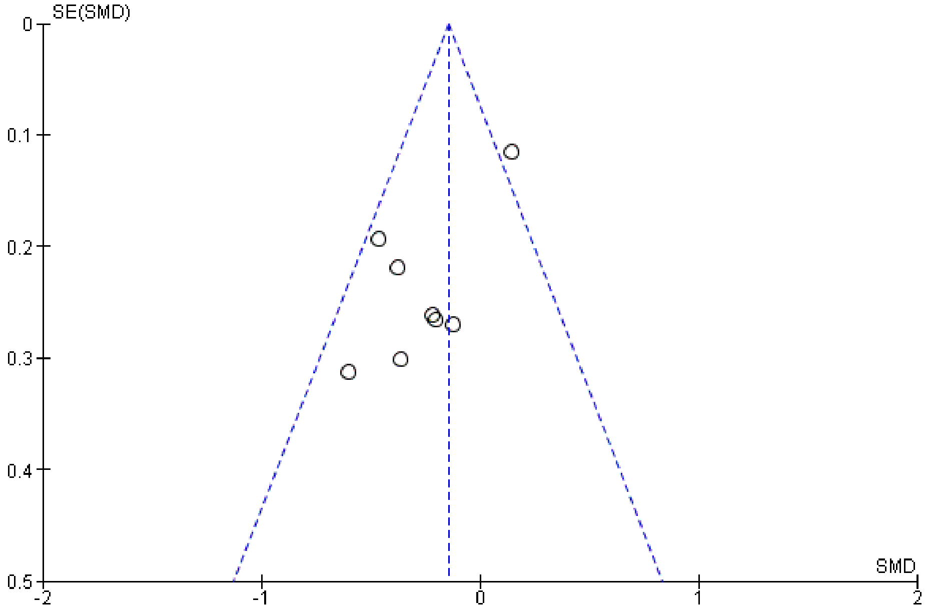

Supplementary Figure 1: Funnel plot for HbA1c

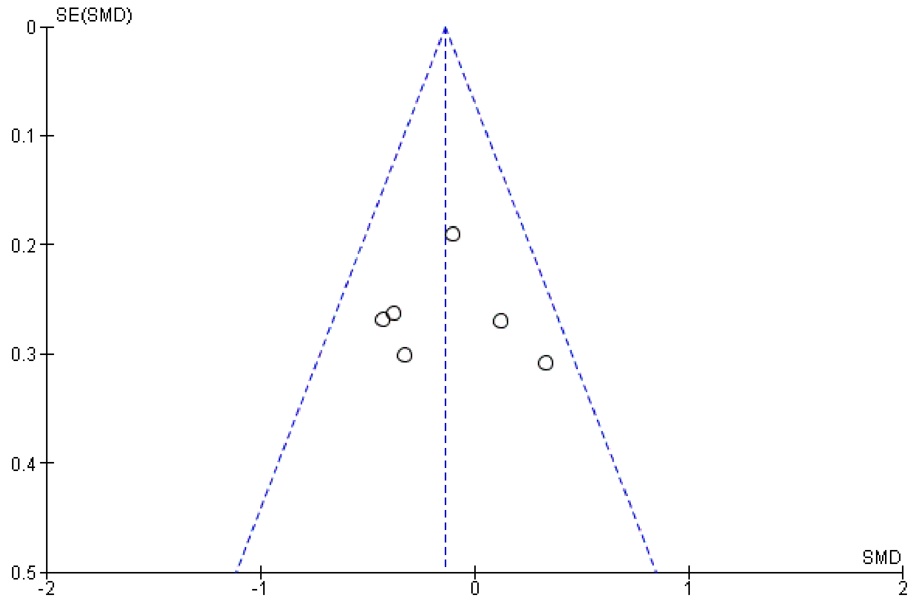

Supplementary Figure 2: Funnel plot for systolic blood pressure

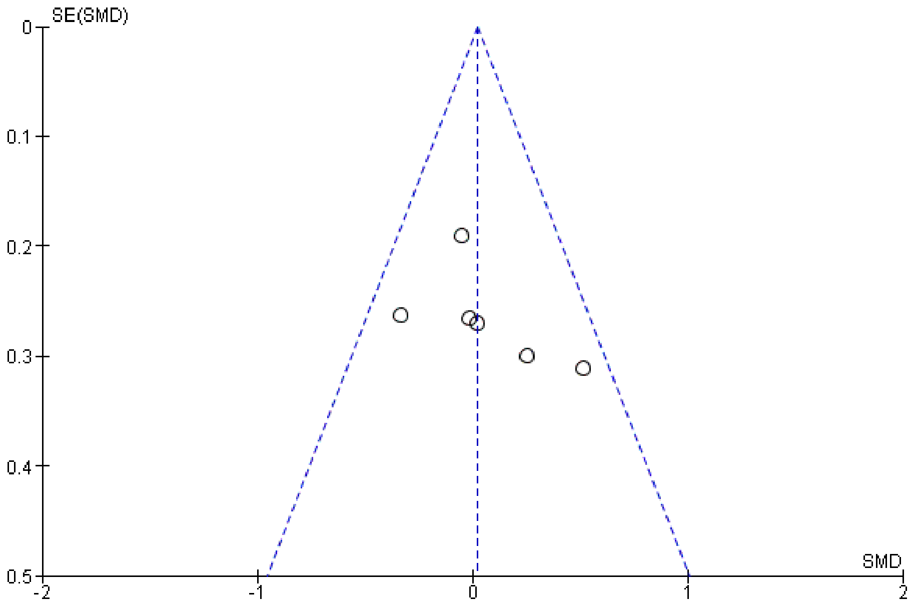

Supplementary Figure 3: Funnel plot for diastolic blood pressure
