# Supplementary material for: Effects of Tocotrienol-Rich Fraction Supplementation in Patients with Type 2 Diabetes: A Systematic Review and Meta-Analysis of Randomized Controlled Trials
Source: Adv Nutr. 2023 Jun 14;14(5):1159–69. doi: 10.1016/j.advnut.2023.06.006 (PMC10509396; doi:10.1016/j.advnut.2023.06.006)
Supplement: Multimedia component2 [file mmc2.pdf]

Title: Effects of tocotrienol-rich fraction supplementation in patients with type 2 diabetes: A systematic review and meta-analysis of randomised controlled trials

First author: Sonia CW Phang

Supplementary Table 1: Full search strategy

No.	Database	Search Terms and Results
1.	PubMed (3 March 2023)	#1: (("tocotrienols"[mh]) OR (tocotrienol[tiab])) OR ("tocotrienol-rich vitamin E" [tiab])) OR ("tocotrienol-rich fraction" [tiab]) OR (annatto [tiab]) OR ("vitamin E" [tiab]))<br><br>#2: (("Type 2 diabetes" [mh] OR diabetes [tiab] OR NIDDM [tiab] OR T2DM [tiab])<br><br>#3: ("randomized controlled trial" [pt] OR "clinical trial" [pt] OR randomized [tiab] OR placebo [tiab])<br><br>Filters applied: Clinical Trial, in the last 10 years, Humans.
2.	OVID Medline (3 March 2023)	#1 INDEXTERMS (tocotrienol OR "tocotrienol-rich fraction" OR "tocotrienol-rich vitamin E" OR "annatto") OR TITLE-ABS-KEY (tocotrienol OR "tocotrienol-rich fraction" OR "tocotrienol-rich vitamin E" OR "annatto")<br><br>#2 INDEXTERMS ("randomized controlled trial" OR "Randomized Controlled Trials as Topic" OR "clinical trial" OR "placebo"))<br><br>#3 INDEXTERMS ("type 2 diabetes" OR diabetes OR NIDDM OR T2DM OR "diabetic neuropathy" [tiab] OR "diabetic peripheral neuropathy" [tiab] OR "diabetic retinopathy" [tiab] OR "diabetic nephropathy")<br><br>#4 #1 AND #2 AND #3<br><br>#5 limit #4 to English language and humans and yr="2012-Current"
3.	Scopus (3 March 2023)	"Type 2 diabetes" OR "Type II diabetes" OR "diabetes mellitus" OR "NIDDM" OR T2DM AND Tocotrienol* OR "tocotrienol-rich fraction" OR TRF OR "tocotrienol-rich vitamin E" OR "vitamin E" OR annatto AND (LIMIT-TO (PUBYEAR , 2023) OR (LIMIT-TO (PUBYEAR , 2022) OR (LIMIT-TO (PUBYEAR , 2021) OR LIMIT-TO (PUBYEAR , 2020) OR LIMIT-TO (PUBYEAR , 2019) OR LIMIT-TO (PUBYEAR , 2018) OR LIMIT-TO (PUBYEAR , 2017) OR LIMIT-TO (PUBYEAR , 2016) OR LIMIT-TO (PUBYEAR , 2015) OR LIMIT-TO (PUBYEAR , 2014) OR LIMIT-TO (PUBYEAR , 2013)

		OR LIMIT-TO (PUBYEAR , 2012) AND (LIMIT-TO (DOCTYPE , "ar")) AND (LIMIT-TO (EXACTKEYWORD , "Human") OR LIMIT-TO (EXACTKEYWORD , "Humans") OR LIMIT-TO (EXACTKEYWORD , "Randomized Controlled Trial (topic)")) AND (LIMIT-TO (LANGUAGE , "English")) AND (LIMIT-TO (SRCTYPE , "j"))
4.	Cochrane Library (3 March 2023)	#1 MeSH descriptor: [Tocotrienols] explode all trees<br>#2 (tocotrienol OR “tocotrienol-rich fraction” OR “tocotrienol-rich vitamin E” OR annatto):ti.ab,kw<br>#3 MeSH descriptor: [Diabetes Mellitus, Type 2] explode all trees<br>#4 #1 OR #2 AND #3
